# Cellular Phenotypic Transformation in Heart Failure Caused by Coronary Heart Disease and Dilated Cardiomyopathy: Delineating at Single-Cell Level

**DOI:** 10.3390/biomedicines10020402

**Published:** 2022-02-08

**Authors:** Luojiang Zhu, Wen Wang, Changzhen Ren, Yangkai Wang, Guanghao Zhang, Jianmin Liu, Weizhong Wang

**Affiliations:** 1 Neurovascular Center, Changhai Hospital, Naval Medical University (Second Military Medical University), Shanghai 200433, China; zhuluojiang@smmu.edu.cn (L.Z.); zhanggh@smmu.edu.cn (G.Z.); 2 Department of Marine Biomedicine and Polar Medicine, Naval Medical Center, Naval Medical University (Second Military Medical University), Shanghai 200433, China; wangwen0120@smmu.edu.cn (W.W.); renchangzhen@smmu.edu.cn (C.R.); wyangkai2005@smmu.edu.cn (Y.W.)

**Keywords:** heart failure, coronary heart disease, single-cell RNA sequencing, phenotypic transformation, myosin, cardiac fibrosis

## Abstract

Heart failure (HF) is known as the final manifestation of cardiovascular diseases. Although cellular heterogeneity of the heart is well understood, the phenotypic transformation of cardiac cells in progress of HF remains obscure. This study aimed to analyze phenotypic transformation of cardiac cells in HF through human single-cell RNA transcriptome profile. Here, phenotypic transformation of cardiomyocytes (CMs), endothelial cells (ECs), and fibroblasts was identified by data analysis and animal experiments. Abnormal myosin subunits including the decrease in Myosin Heavy Chain 6, Myosin Light Chain 7 and the increase in Myosin Heavy Chain 7 were found in CMs. Two disease phenotypes of ECs named inflammatory ECs and muscularized ECs were identified. In addition, myofibroblast was increased in HF and highly associated with abnormal extracellular matrix. Our study proposed an integrated map of phenotypic transformation of cardiac cells and highlighted the intercellular communication in HF. This detailed definition of cellular transformation will facilitate cell-based mapping of novel interventional targets for the treatment of HF.

## 1. Introduction

Heart failure (HF) is a complex disease, which represents the final stage for several heart diseases, such as coronary heart disease and various cardiomyopathies. Despite decades of research and the development of multi-modality treatments, HF remains a major and growing public health problem worldwide due to poor heart regeneration after injury. Recent studies have revealed the cellular diversity in cardiac physiological and pathophysiological conditions with the rapid development of single-cell techniques [1,2]. Richard et.al mapped the origin of the embryonic mouse heart with single-cell transcriptomic, providing a transcriptional and anatomic definition of cardiac progenitor types and revealing a cardiac progenitor pool which was a source of trophic factors and cells during cardiac development and injury [3]. Furthermore, scRNA-seq was applied to distinguish the T cell type in the heart with myocardial infarction or myocardial ischemia/reperfusion injury and a phenotypically and functionally unique population of heart-regulated T cells was identified, which exert heart protective effects in many diseases [4]. These studies revealed the great potential of scRNA-seq in exploring the mechanism of heart failure, which will make cell therapy or cell transplantation a promising approach for restoring cardiac function after heart failure. Here, we used scRNA-seq to explore the unclear interaction and transformation among different types of cardiac cells in HF.

Cellular phenotypic transformation has been considered as an important role in many chronic cardiovascular diseases, such as vascular smooth muscle cell and macrophage in atherosclerosis [5,6]. Many studies have demonstrated that cell transformation occurred in HF as well, for example, fibroblasts (FBs) became hypertrophic and secrete collagen, which promoted myocardial fibrosis [7], underlining the need to study phenotypic transformation in HF in detail. Furthermore, abnormal expression of myosin was confirmed in HF. Myosin is one of the most critical contractile proteins in cardiomyocytes (CMs), composed of two myosin heavy chains (MHC) and four myosin light chains (MLC) [8]. Generally, MHC is mainly divided into two subtypes, Myosin Heavy Chain 6 (*MYH6*) and Myosin Heavy Chain 7 (*MYH7*), which constitute the body of myosin [9]. MLC can be divided into essential light chain (ELC) and regulatory light chain (RLC) [10,11]. Limited by previously technology, little was known about the functional shift and cellular transformation of each cardiac cell during the progression of HF, which hampers the deeper understanding of HF. Based on recent research focusing on cellular diversity in human and rat hearts through scRNA-seq technology, it is suggested that further investigation of phenotypic transformation can better explain the progress of HF.

Here, we analyzed the transformation of phenotype in the human heart tissue with HF caused by coronary heart disease (cHF) or dilated cardiomyopathy (dHF) using scRNA-seq, mainly including CMs, endothelial cells (ECs), and FBs. Multiple bioinformatics tools and the HF rat model were applied, and we finally confirmed the features of cellular phenotypic transformation in HF (including cHF and dHF) as well as proposed an integrated map of microenvironmental changes, which may lead to cardiac remodeling and reduced myocardial contractility. Our study provided new insights for the occurrence and development of HF, which may be helpful to develop new treatments targeting on myosin and inhibition of myocardial fibrosis.

## 2. Materials and Methods

### 2.1. Single-Cell Dataset Download and Quality Control

The single-cell dataset (GSE121893 and GSE109816) was downloaded from the Gene Expression Omnibus (GEO), including 2 cHF, 5 nHF, and 3 dHF human heart tissue. The logarithmic value of Unique Molecular Identifiers (UMI) in the download matrix was normalized to obtain the corresponding Transcripts Per Million (TPM) value so as to standardize sequencing depth and gene length. Genes expressed in at least three cells and eliminated cells with nFeature_RNA less than 200 or greater than 5000 were obtained. Mitochondrial genes were deleted.

### 2.2. Dimension Reduction and Clustering

We used the Seurat V4.01 function package in software R V4.02 to integrate the above filtered matrix [12]. The functions FindIntegrationAnchors and IntegratedData were used to integrate data. The Harmony algorithm was used to eliminate batch effects [13]. We performed principal component analysis (PCA) on highly variable genes, then visualized the cells through the Unified Manifold Approximation and Projection (UMAP) algorithm [14]. Finally, we annotated five main cell groups.

### 2.3. Cell Trajectory Analysis

The Monocle3 V0.22 algorithm was used to calculate the trajectory of CMs [15]. The cell-gene expression matrix of CMs was transformed into cds object. The differentiation trajectory was deduced by Learn_graph function. The Order_cell function is used to pseudotime analysis.

### 2.4. Differentially Expressed Genes (DEGs) Display

Functions FindAllMarkers are used to calculate DEGs among multiple cell clusters. We used the Top_n function to extract the five most significant genes in each cluster and displayed them with a Heatmap or Bubble diagram. In addition, the ggplot2 function was used to draw volcano, histograms, and fiddle diagrams to specifically display the genes.

### 2.5. Gene Set Variation Analysis (GSVA)

The integrated cell-gene expression matrix was taken as the input file. GO and KEGG related pathways were taken as reference file. The pathway enrichment in each cell was calculated by GSVA V1.36.3 algorithm [16], the cell gene set (pathway) expression matrix was obtained finally. Reference file were downloaded in website (http://www.gsea-msigdb.org/gsea/downloads.jsp accessed on 25 July 2021).

### 2.6. Transcription Factors (TFs) Activity Analysis

Single-Cell Regulatory Network Inference and Clustering (SCENIC, V1.13) algorithm was applied [17]. We calculated the regulatory factors (TFs and its target genes) in each cell. RcisTarget database was used as reference file. Genie3 and Spearman were used to calculate the correlation between the transcription factor and the potential target. Then generated GRNs (also known as regulons) through the “RunScenic” program. Finally, the activity score of regulons was analyzed by Area Under the Curve (Aucell) function to obtain the cell-TF matrix.

### 2.7. Cellular Communication Computing

CellPhoneDB V1.36.3 software systematically analyzed the communication molecules between cells [18]. The list of receptor-ligand pairs can be found at https://www.cellphonedb.org/downloads. We downloaded them on 25 August 2021. Counts quantitative data and cell type annotation information are used as input files. The receptor-ligand pair was included in the analysis only when the percentage of cells expressing the receptor and ligand genes exceeded a specified threshold (the default was 10%). Finally, the significant P values of the receptor-ligand pair in each two cell types were deduced by the algorithm analysis.

### 2.8. Gene Set Enrichment Analysis (GSEA)

GEO dataset (GSE42955) was applied in GSEA including 5 normal hearts, 12 diluted cardiomyopathies, and 12 ischemic cardiomyopathies. R software and related package were used. The DEGs between normal hearts and diluted cardiomyopathy or ischemic cardiomyopathy were firstly calculated. Then the GSEA function package was used to analyze. Reference file “c5.bp.v7.0.symbols.gmt” were download from GSEA website (http://www.gsea-msigdb.org/gsea/downloads.jsp accessed on 20 January 2022). The results were visualized using gseaplot2.

### 2.9. Establishment of Heart Failure Model in Rats

Adult male Sprague Dawley rats (16–18 weeks, weight 280–350 g) were purchased from the Naval Medical Center (Shanghai, China). They were divided into the HF group and the sham group randomly. All experimental rats were placed in animal rooms with 12 h of light and 12 h of darkness, having free access to water and feed. The rats were anesthetized using sodium pentobarbital (40 mg/kg) by intraperitoneal injection. Rats in the HF group were ligated with the anterior descending branch of the left coronary artery, and the chest was closed after the heart turned purple, which is the model similar to the clinical heart failure caused by ischemic heart disease [19]. The sham group performed the same operation except ligation of blood vessels. As described in our previous work [20], the cardiac function of rats in the HF group and the sham group was evaluated by echocardiography 28 days after operation. Briefly, rats were anesthetized with continuous inhalation of 2% isoflurane and transthoracic echocardiography was performed using a high-resolution ultrasound imaging system (Vevo 2100 Linear Imaging System, FUJIFILM Visual Sonics, Toronto, Canada). The M-mode recordings are from the parasternal short axis view. Left Ventricular Systolic Posterior Wall Thickness (LVPWs), Left Ventricular Systolic Inner Diameter (LVIDs), End Diastolic Volume (EDV), Left Ventricular Diastolic Posterior Wall Thickness (LVPWd), Left Ventricular Diastolic Inner Diameter (LVIDd), End-Systolic Volume (ESV), Fractional Shortening (FS), and Ejection Fraction (EF) were used to evaluate cardiac function. After evaluating cardiac function, the rats were sacrificed. The hearts were isolated and frozen in liquid nitrogen for subsequent experiments.

### 2.10. Immunohistochemical and Immunofluorescence

The 5μm slice of heart tissue was incubated overnight with *Myh6* (proteintech, 22281-1-AP,1:100, Beijing, China), and *Myl7* (proteintech, 17283-1-AP, 1:200, Beijing, China) antibodies at 4 °C. After washing for 3 times with PBS, the sections were incubated with HRP-labeled goat anti-rabbit secondary antibody (ServiceBio, GB23303, 1:200, Wuhan, China) at 37 °C for 50 min. The images were taken using a light microscopy (Olympus, Japan). ImageJ software was applied to quantify the expression of *MYH6* and *MYH7* in immunohistochemical staining. Here, we used AOD (Average Optical Density) to quantify the immunohistochemical results. AOD = IOD/Area. IOD represented the value of integrated option density of the graph which was proportional to the total amount of target protein. As for immunofluorescence, Rabbit *Myl7* antibody (proteintech, 17283-1-AP, 1:200, Beijing, China) or Rabbit *Myh6* antibody (proteintech, 22281-1-AP, 1:200, Beijing, China), and Mouse *Pecam1* antibody (ServiceBio, GB12063, 1:200, Wuhan, China) are used for double immunostaining in ECs. Mouse Vimentin antibody (ServiceBio, GB12192, 1:500, Wuhan, China) and Rabbit *Actn2* antibody (ServiceBio, GB11555, 1:500, Wuhan, China) are used for double immunostaining in FBs. Secondary antibodies included CY3-labeled goat anti-mouse (ServiceBio, GB21301, 1:300, Wuhan, China) and goat anti-rabbit (ServiceBio, GB25303, 1:400, Wuhan, China) are used. Sections were observed and images were collected under a fluorescence microscope (Nikon, Nikon Eclipse C1, Tokyo, Japan).

### 2.11. Total RNA Extraction and Real Time-PCR Analysis

Total RNA was extracted from the HF rats and the sham group using Trizol reagent (Invitrogen, CA, USA) and 0.5 μg RNA was reverse-transcribed (Takara, Otsu, Shiga, Japan). Quantitative real-time PCR was performed with TB Green**^®^** Premix Ex Taq™ II (Takara, Otsu, Shiga, Japan) with 2 μL cDNA and specific primer encoding GAPDH was measured as a reference gene.

### 2.12. Masson Staining and Fibrosis Analysis

After dewaxing and rehydrating, the sections were stained with hematoxylin for 10 min and rinsed with double-distilled water. Lichun red acid magenta is stained for 10 min at room temperature, followed by an additional wash with water. The solution was differentiated with 1% phosphomolybdate solution for 5 min, immediately followed by dyeing with aniline blue or light green solution for 5 min. After washing with 0.2% glacial acetic acid, the samples were dehydrated in gradient and sealed with neutral gum. The collagen fibrils were analyzed by Image-Pro Plus 6.0 software. The detail steps are as follows. Firstly, the Masson staining picture was put into ImageJ software. Then, adjust the threshold such as hue, saturation, and brightness to locate the positive area. The fibrosis area was turned to red and then selected. Finally, the red area was figured out and measured.

### 2.13. Statistical Analysis

Data are presented as mean ± SEM unless otherwise stated. Comparison of the two groups was examined by unpaired Student’s *t*-test. Multiple group comparisons were made by one-way ANOVA followed by the Bonferroni test. The chi-square test was used to calculate the difference between two or more rates (component ratios). The difference was considered significant at *p* < 0.05. The default Wilcox test is used for statistical tests in the single-cell algorithm. The difference was considered significant at Adjusted *p* value (Padj) < 0.05. Statistical analysis was performed using GraphPad Prism 8 software.

## 3. Results

### 3.1. Single-Cell Profile of Normal and Failure Heart Tissue

**Figure 1:**
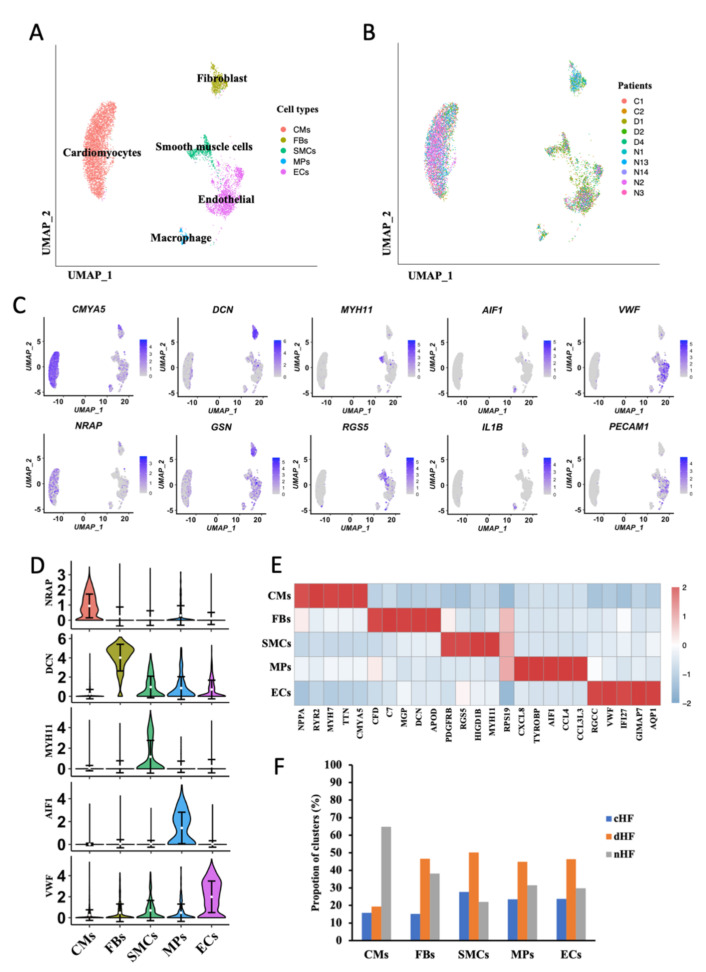
Five cell types were identified from single-cell transcriptome of heart disease. (**A**,**B**) Uniform Manifold Approximation and Projection (UMAP) plots. Clusters are color-coded by cell types (**A**) and patient (**B**). (**C**) Canonical markers for 5 different cell clusters on the UMAP plot. (**D**) Violin plots of canonical marker of 5 cell types. (**E**) Heatmap depicting the top 5 differentially expressed genes (DEGs) in each cell cluster. Rows indicate cell types and columns indicate genes. (**F**) Cell proportion of 3 sample sources in each cell type.

Single-cell sequencing data were collected from the GEO database which was composed of 10 human cardiac tissue samples, including HF caused by coronary heart disease (cHF), dilated cardiomyopathy (dHF), and normal tissues (nHF). All cells from all the patients were divided into five groups. including CMs, ECs, FBs, Macrophages (MPs), and Smooth Muscle Cells (SMCs) (Figure 1A,B). The five cell groups were identified with canonical genes, as CMYA5, NRAP for CMs, DCN, GSN for FBs, MYH11, RGS5 for SMCs, AIF1, IL1B for MPs and VWF, PECAM1 for ECs (Figure 1C), and the expression of canonical markers for each group was exhibited (Figure 1D). All kinds of clusters differ in their molecular markers (Figure 1E). Cells were then colored by three samples (cHF, dHF, and nHF) (Supplementary Figure S1A). It is found that CMs and ECs occupied the most part in each sample (Supplementary Figure S1B), and each sample had the same cell groups (Supplementary Figure S1C). The cell proportion of three kinds of samples in five clusters were showed in Figure 1F. With the consideration of cell composition in each cluster, we noticed that CMs, ECs, and FBs made up the majority of the total cells while the number of SMCs and MPs was insufficient. In addition, plenty of studies mentioned that CMs, ECs, and FBs played pivotal roles in HF progress. In order to avoid false positive results and grasp the core mechanism in HF, we finally analyzed the phenotypic transformation of CMs, ECs, and FBs.

### 3.2. Trajectory Analysis Disclosure the Transcriptional Dynamics of CMs during Progress of HF

**Figure 2:**
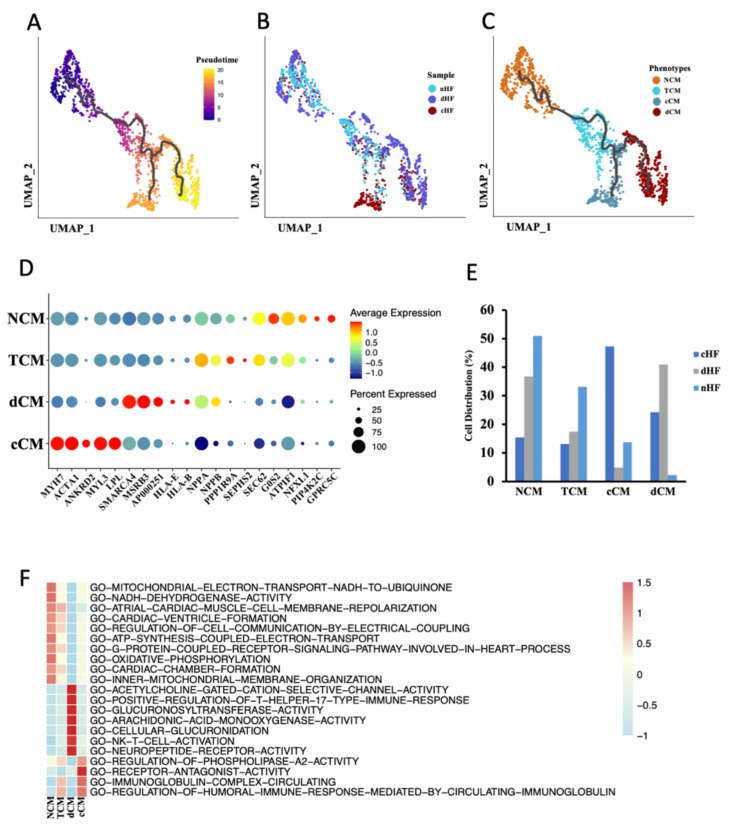
Cardiomyocytes (CMs) cluster into 4 distinct phenotypes. (**A**–**C**) Cell trajectory of CM calculated by Monocle3, cells were colored by pseudotime (**A**), sample (**B**), and phenotypes (**C**). (**D**) Dot plot of CM subpopulations demonstrates the top five markers of each type. Dot size corresponds to proportion of cells expressing each transcript within the group, and dot color stands for scaled expression level. (**E**) The distribution of three kinds of samples (nHF, cHF, dHF) in four CM phenotypes (NCM, ECM, cCM, dCM). (**F**) Differences of pathway enrichment among 4 CM phenotypes.

To understand the transcriptional dynamics that occurred in CMs, pseudotime trajectory analysis was performed to predict continuous cell states in CMs. In order to avoid heterogeneity of CMs between the ventricle and atrium, we only used the mixed sample of LA and LV (GSE121893) for further analysis. According to the pseudotime trajectory, there were two differentiation directions in CMs (Figure 2A). The distribution of CMs derived from different sample sources was further shown in the trajectory (Figure 2B). According to the distribution and trajectory, CMs were then clustered into four phenotypes, named Normal Cardiomyocytes (NCM), Transient Cardiomyocytes (TCM), cHF Cardiomyocytes (cCM), and dHF Cardiomyocytes (dCM) (Figure 2C). TCM was the intermediate stage in phenotypic transformation of CMs according to its similarity of gene expression pattern and pathway enrichment with NCM. In order to confirm the above conjecture, top genes of ordered cells were then revealed, as well as cell composition in each phenotype. Cell type-specific markers for a phenotype was defined as the genes with the highest differential expression relative to all other phenotypes (Figure 2D and Supplementary Figure S2A). The ordinate of the chart (Figure 2E) showed the distribution of CMs derived from cHF, dHF, and nHF in the four phenotypes, respectively. These results firmly validate the reliability of our identification of the four phenotypes.

In order to map functional and transcriptional changes in HF, gene set variable analysis (GSVA) was applied to calculate the pathway enrichment score. Each phenotype had a type-specific pathway, respectively (Figure 2F). TCM had similar pathway enrichment with NCM, along with a lower enrichment score, this may attribute to the transient condition of TCM. In addition, compared to NCM, cCM and dCM shared the same trends of some pathways, but there were still huge differences between these two phenotypes. It was noticed that both cCM and dCM were involved in downregulated pathways about energy metabolisms, such as GO-OXIDATIVE-PHOSPHORYLATION and GO-MITOCHONDRIAL-ELECTRON-TRANSPORT-NADH-TO-UBIQUINONE, while dCM had lower enrichment score than cCM (Supplementary Figure S2B). Compared with NCM, dCM showed a higher reaction to T cells because pathways such as GO-POSITIVE-REGULATION-OF-T-HELPER-17-TYPE-IMMUNE-RESPONSE and GO-NK-T-CELL-ACTIVATION were upregulated in dCM, while cCM made no difference. (Supplementary Figure S2B). Another dataset from the GEO database (GSE42955) was applied to re-confirm functional and transcriptional changes in HF. This dataset composed of human heart tissue from 29 individuals, including 5 normal hearts, 12 diluted cardiomyopathies, and 12 ischemic cardiomyopathies. The results of the GSEA suggested that compared with the normal hearts, ischemic cardiomyopathy significantly downregulated GO-OXIDATIVE-PHOSPHORYLATION as well as GO-MITOCHONDRIAL-ELECTRON-TRANSPORT-NADH-TO-UBIQUINONE. Although GSEA results didn’t indicate these two pathways highly expressed in diluted cardiomyopathy, we found that GO-T-CELL-ACTIVATION and GO-REGULATION-OF-AUTOPHAGE-OF-MITOCHONDRION were upregulated in diluted cardiomyopathy (Supplementary Figure S2C). These results demonstrated that ischemic and diluted hearts had lower energy metabolism function, and T cells in diluted cardiomyopathy were highly activated, which were consistent with our previous conclusion. Taken together, these results uncovered transcriptional and function dynamics of CMs in HF. Abnormal energy metabolism was existing in CMs of HF, both in cHF and dHF, while dCM had a specific response to T cells.

### 3.3. Transformation of MHC and MLC in cCM

**Figure 3:**
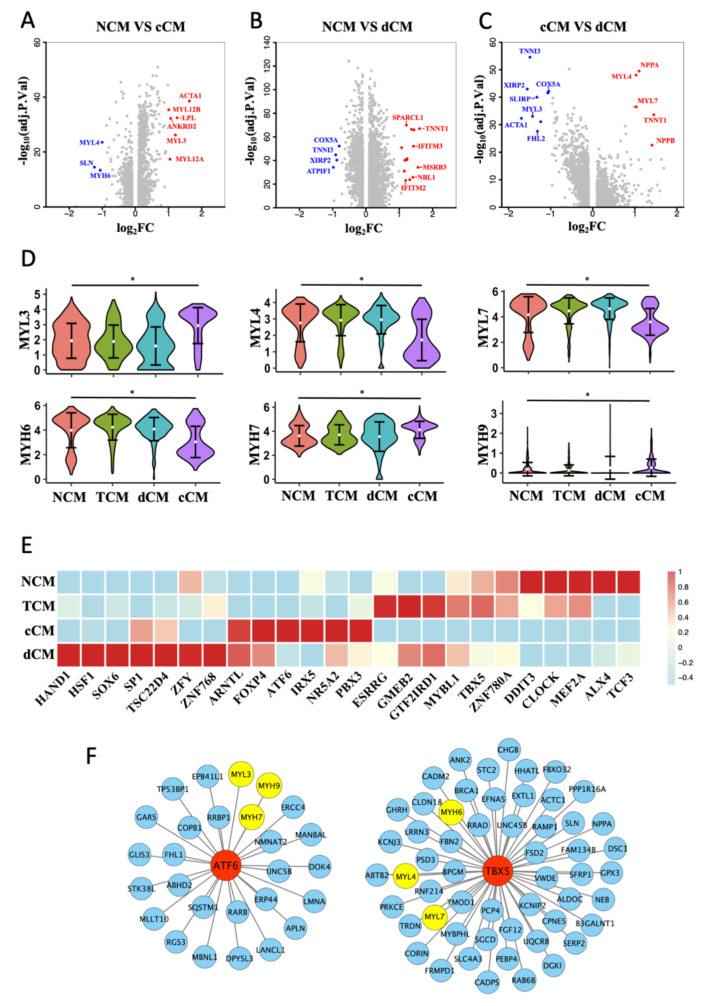
Abnormal expression of myosin subunits in cCM. (**A**–**C**) Volcano plot of DEGs (log2FC > 1) between each 2 CM phenotypes. (**D**) Violin plots show the scaled expression of different subtypes of MLC and MHC in 4 CM phenotypes. * Padj < 0.05, Wilcox test. (**E**) Heat map of phenotype-specific TFs activity in 4 CM phenotypes. (**F**) TBX5 and ATF6 regulates downstream genes predicted by SCENIC.

After comparing DEGs between NCM, cCM, and dCM (Figure 3A,C), we noticed that genes encoding subunits of myosin had different expressions between NCM and cCM (Figure 3D). It is found that MYH6 was significantly downregulated, while the expression of MYH7 was obviously increased in cCM. The expression of MLC showed that the expressions of three main subtypes, MYL3, MYL4, and MYL7 were changed in cCM (Figure 3D). The MYL3 decreased significantly in cCM while MYL4 and the RLC (MYL7) were obviously downregulated. Interestingly, there was no significant difference in MHC and MLC between dCM and NCM (Figure 3D), which meant that dHF may differ from cHF in pathological mechanisms.

SCENIC algorithms were further used to calculate the activity of TFs in the four phenotypes of CMs. Specific TFs in each phenotype were shown in a heatmap (Figure 3E). After predicting the downstream genes of phenotype-specific TFs, two TFs, TBX5 and ATF6, were picked out, which were highly expressed in NCM and cCM, respectively. The prediction results indicated that ATF6 could regulate MYL3, MYH9, and MYH7, while TBX5 could regulate MYL4, MYH6, and MYL7 (Figure 3F).

**Figure 4:**
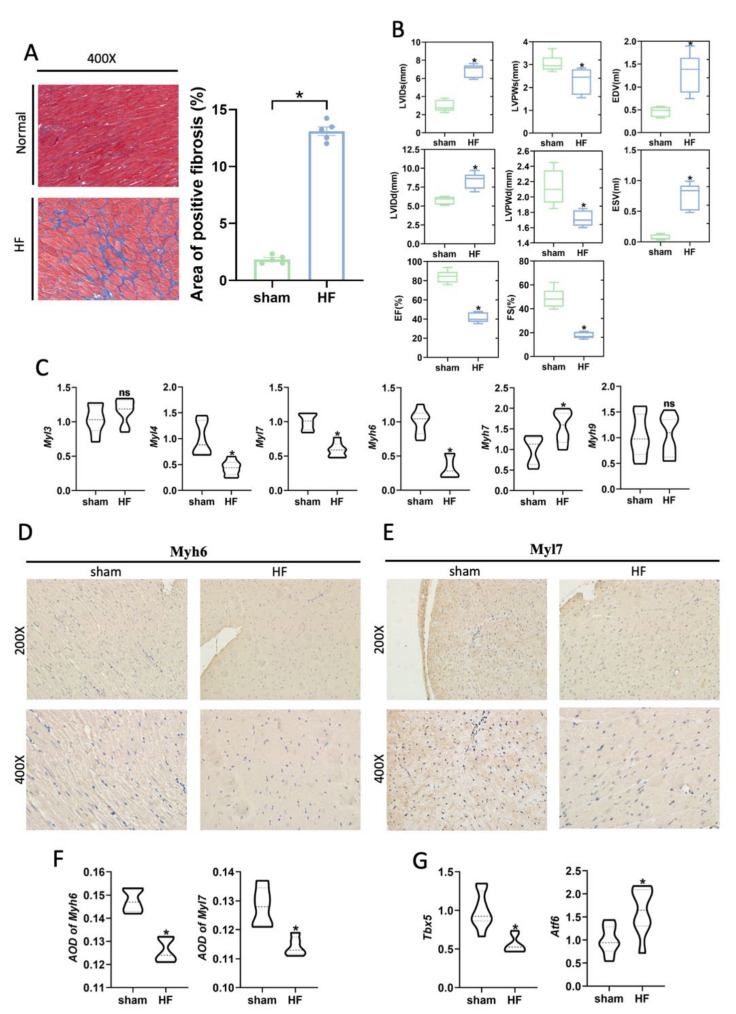
Abnormal expression of myosin subunits was supported in the rat heart failure models. (**A**) Masson staining of the HF and sham group (**Left**). Histogram of the area of positive fibrosis (**Right**). *n* = 5 per group. * *p* < 0.05 vs. sham. (**B**) Left ventricular systolic function parameters images at the study endpoint between the HF and sham group. *n* = 5 per group. * *p* < 0.05 vs. sham. LVIDd: Left ventricular diastolic inner diameter; LVEF: Left ventricular ejection fraction; LVIDs: Left ventricular systolic inner diameter; FS: Fraction shortener; EF: Ejection Fraction; LVPWd: left ventricular diastolic posterior wall thickness; LVPWs: Left ventricular systolic posterior wall thickness. (**C**) mRNA expression of MHC and MLC subunits. *n* = 8 per group. * *p* < 0.05 vs. sham; ns: no significance. (**D**,**E**) Representative immunohistochemistry images of the hearts of the sham and HF group indicate the myosin subunits change, including Myl7, Myh6. (**F**) AOD of Myh6 or Myl7 immunohistochemistry images. *n* = 5 per group. * *p* < 0.05 vs. sham. (**G**) mRNA expression of transcription factors TBX5 and ATF6, *n* = 8 per group. * *p* < 0.05 vs. sham.

The majority of cHF is resulted from myocardial ischemia caused by coronary artery stenosis. A heart failure rat model after myocardial infarction, which was a good representation of cHF, was then constructed to validate the above results. Compared with the sham group, the myocardial fibrosis area of the ventricular wall was obviously increased (Figure 4A). Cardiac function, which was evaluated by ultrasonography, obviously showed impaired myocardial contractibility, and reduced ejection fraction in HF rats (Figure 4B). Consistent with the results of our previous analysis, the expression of MYL4, MYL7, and MYH6 was decreased in the HF group, while the expression of MYH7 was increased (Figure 4C). Notably, MYL3 was not significantly increased in the HF group. Immunohistochemical staining revealed an obvious decrease in expression of MYL7 and MYH6 in the HF group (Figure 4D,E). The AOD of immunohistochemical staining significantly indicated that the HF group had lower expression of MYH6 and MYL7 compared with the sham group (Figure 4F). In summary, these results demonstrated that CMs had phenotypes transformation in cHF, especially the changes in myosin, which may lead to the decline of myocardial contractility. Additionally, it was confirmed that related transcription factor, TBX5 and ATF6, were highly expressed in the sham and HF group, respectively (Figure 4G).

### 3.4. Two Special Pathological Phenotypes of ECs in HF

**Figure 5:**
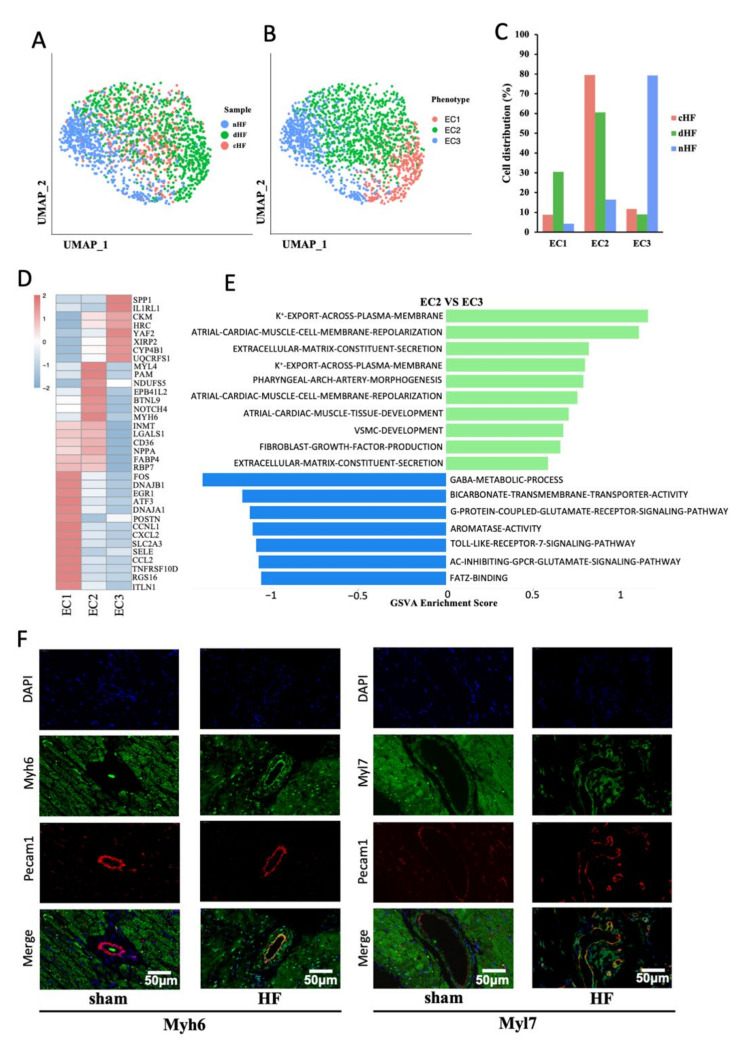
Endothelial cells in HF have two special disease-related phenotypes. (**A**, **B**) UMAP plot of ECs, subgroups were colored by phenotypes (**A**) and sample s (**B**). (**C**) The distribution of cells from nHF, cHF, and dHF in three phenotypes (EC1, EC2, EC3). (**D**) The heatmap shows the differential gene expression pattern of three phenotypes of ECs. (**E**) Difference of pathway enrichment between EC1 and EC3. (**F**) Immunofluorescence showed the presence of myosin subunits (Myh6, Myl7) in ECs of heart vessels in the HF (right).

We then focused on the phenotypic transformation of ECs in HF. According to the original article [2], no heterogeneity existed in ECs between ventricle and atrium, which justifies our use of data to analyze ECs. ECs were divided into three subsets by performing dimensionality reduction clustering, named EC1, EC2, and EC3 (Figure 5A,B). From the cell distribution of samples, we noticed that most ECs from cHF were located in EC2, and ECs from dHF were in EC1 and EC2, while ECs from nHF were in EC3 (Figure 5C). The heatmap of three phenotypes showed that each phenotype had a special gene expression pattern (Figure 5D), which suggested that they may play different roles in the progression of HF. Given the gene expression pattern and cell distribution of three phenotypes, EC1 was considered as an inflammation phenotype, EC2 was a muscularized phenotype, and EC3 was a normal phenotype. We then calculated and displayed the specific genes in each phenotype (Supplementary Figure S3A,B).

EC1 was an inflammation-related endothelial cells that can secrete CCL2, CXCL2, and other chemokines, attracting immunity cell infiltration. Pathway enrichment showed that EC1 was highly expressed in inflammation-related pathways, such as GO-POSITIVE-REGULATION-OF-TNF-MEDIATED-SIGNALING-PATHWAY and GO-REGULATION-OF-MONOCYTE-CHEMOTAXIS (Supplementary Figure S3C). At the transcriptional level, EC1 was also found to highly expressed inflammatory TFs such as Jun and Fos [21,22] (Supplementary Figure S3D). The diverse functional involvement of ECs prompted us to explore cell–cell communications of EC1, the crosstalk showed that EC1 could interact with other cells through many chemokines and receptors (Supplementary Figure S3E). Taken together, this evidence suggested that EC1 might play an important role in immune microenvironment development in HF, especially in dilated cardiomyopathy.

Interestingly, EC2 highly expressed marker genes of CMs, such as MYH6, MYL4, and MYL7 (Figure 5D and Supplementary Figure S3A). Pathway analysis also showed that EC2 was enriched with some special pathways such as GO-ATRIAL-CARDIAC-MUSCLE-CELL-MEMBRANE-REPOLARIZATION and GO-VSMC-DEVELOPMENT (Figure 5E). Therefore, it was possible that a transformation of ECs to CMs may exist in HF, and we named them muscularized ECs. Immunofluorescence staining of MYH6 and MYL7 in the heart from the HF rat model presented colocalization with PECAM1, which stood for the presence of myocyte specific muscle fibers in the endothelial cells (Figure 5F). These results indicated that the transformation from ECs to CMs happened in vascular endothelial cells of HF patients, which may affect the myocardial blood supply and aggravate the myocardial systolic disorders.

### 3.5. Myofibroblast Was Increased in Heart Failure

**Figure 6:**
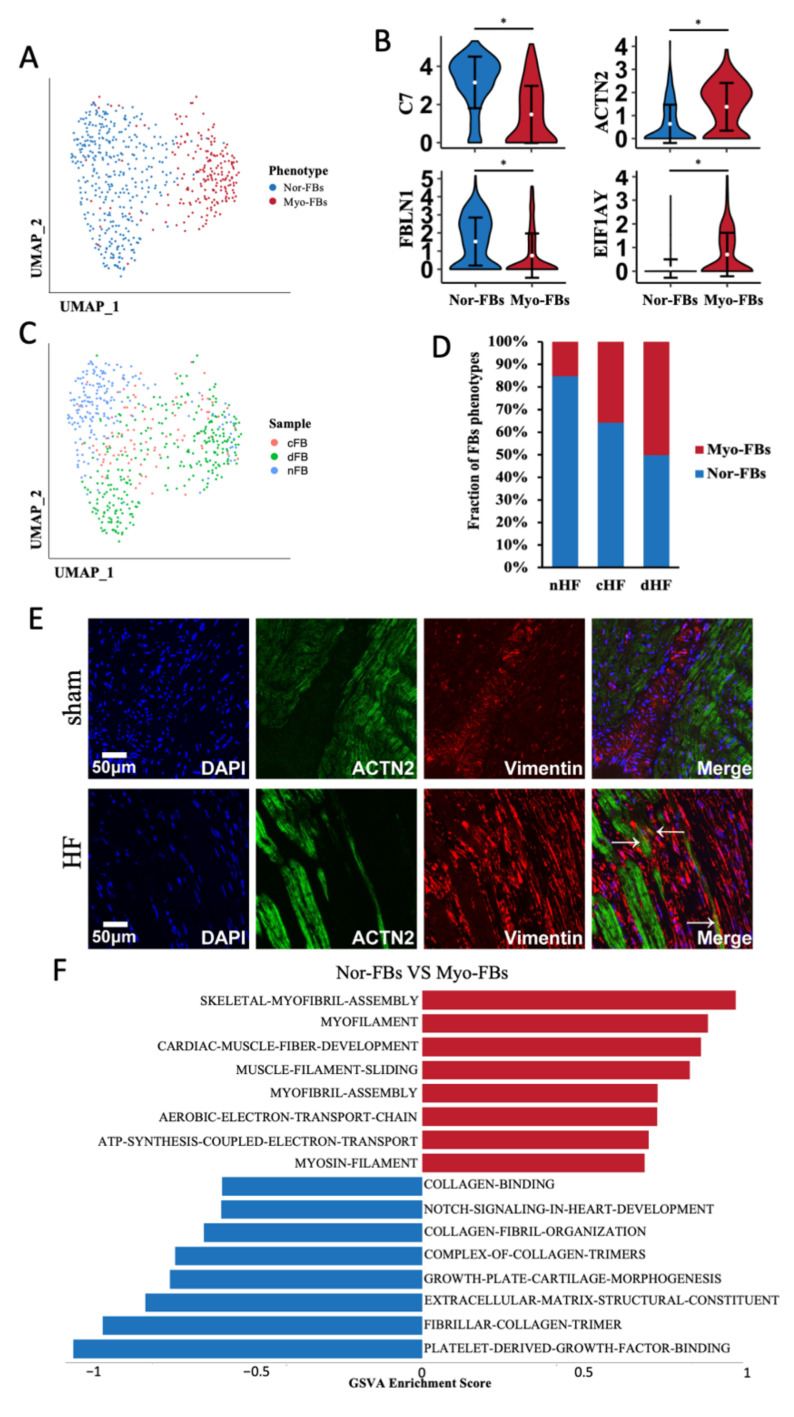
Fibroblasts transformed into Myofibroblasts. (**A**) UMAP plot of FBs, clusters were colored by phenotypes, Nor-FBs and Myo-FBs. (**B**) Violin plots show the different markers of Nor-FBs and Myo-FBs. * Padj < 0.05 Wilcox test. (**C**) Cell distribution of three sample sources in two phenotypes. (**D**) Proportion of Myo-FBs in cHF and dHF were more than nHF. (**E**) Immunofluorescence showed the colocalization of Vimentin and ACTN2 in the HF group. (**F**) Differential pathway enrichment between Nor-FBs and Myo-FBs.

Two phenotypes of FBs were identified according to the dimension reduction analysis (Figure 6A). Given the DEGs between two phenotypes of FBs (Supplementary Figure S4A), we named them as normal fibroblasts (Nor-FBs) and myofibroblasts (Myo-FBs). Compared with Nor-FBs, the expression of canonical markers in Myo-FBs such as C7 and FBLN1 was significantly reduced, while the expression of muscle proteins such as ACTN2 and EIF1AY was increased (Figure 6B). After calculating the cell distribution of two phenotypes in three samples, it was found that the proportion of Myo-FBs in cHF and dHF was much higher than that in nHF (Figure 6C,D). Colocalization of Vimentin and Actn2 was applied to verify this transformation. Vimentin represented the FBs and Actn2 was a kind of alpha-actinin marked for Myo-FBs. It was found that FBs were proliferated in the site of myocardial infarction and Actn2 was colocalized with Vimentin (Figure 6E). This evidence identified the transformation of FBs into Myo-FBs. Myo-FBs were highly enriched of some muscle-related pathways (Figure 6F). In addition, TBX5, the potential transcription factor of some myosin genes was highly activated in Myo-FBs (Supplementary Figure S4B). Above all, we demonstrated that the phenotypic transformation from Nor-FBs to Myo-FBs occurs during heart failure.

To explore the functional change between Nor-FBs and Myo-FBs, we calculated the crosstalk of Nor-FBs and Myo-FBs (Supplementary Figure S4C), the receptor-ligand pair information between Myo-FBs and other cells showed that the ability of Myo-FBs to secrete extracellular matrix was greatly reduced. In either cHF or dHF, the Myo-FBs secreted less collagen to combinate with CMs. In summary, in HF, FBs underwent Myo-FBs transformation, disorderly form extracellular matrix, which was considered as an important factor in myocardial fibrosis.

### 3.6. Intercellular Communication Reveals the Microenvironment cHF

**Figure 7:**
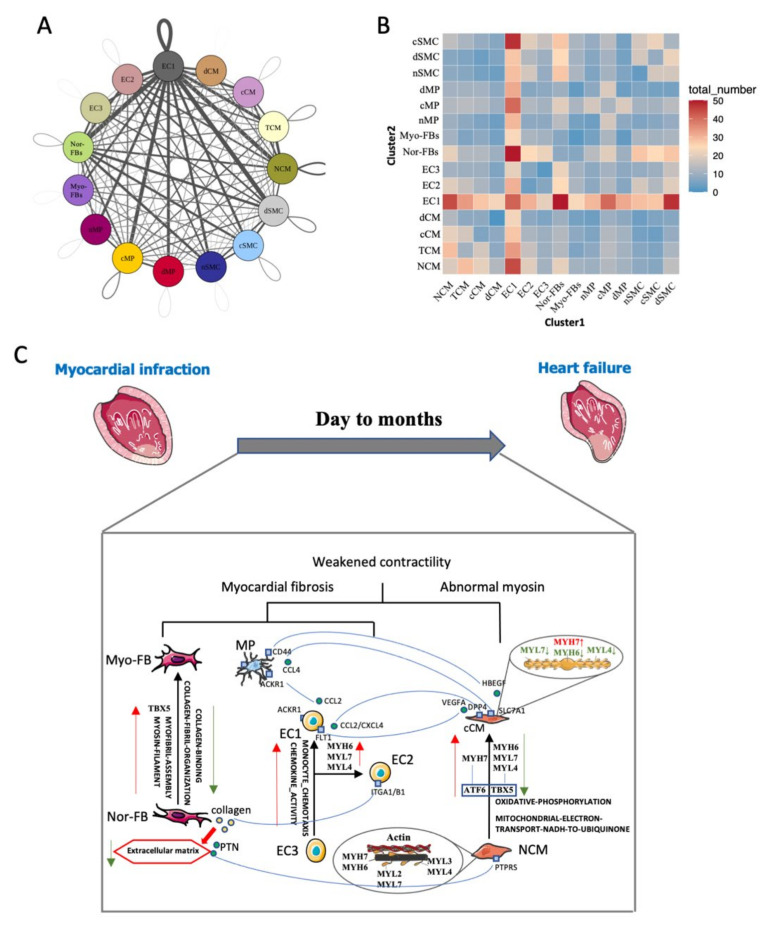
Intracardiac regulatory network-mediated microenvironmental changes in HF. (**A**) Receptor–ligand pairs between cell populations. Each point represented one cell group; the size of the point indicated the number of receptor-ligand pairs associated with it. Lines represented the pairs between two groups, the thicker the line is, the more pairs exist. (**B**) Heatmap shows the number of interactions between two cell clusters (**C**) Network of potential mechanisms from myocardial infarction to heart failure.

Changes in the microenvironment in HF may cause for the progression of heart failure. Therefore, the communication network was constructed. The results showed that there were complex connections among various cell populations (Figure 7A). The number of ligand-receptor pairs indicated that EC1 played an active role in HF (Figure 7B). As we all know, myocardial fibrosis and decreased contractility of CMs were the most important reasons for cHF. In our study, we demonstrated that phenotypic transformation occurred in cHF, and different subsets can interact with others to form a microenvironment of cHF. The crosstalk reveals that cCM can secret VEGFA, binding to FLT1 in inflammatory ECs (EC1), which contributed to angiogenesis. Additionally, cCM can attract macrophage through HBEGF-CD44 pair which had the function of promoting an immune response. ECs had two pathology phenotypes and displayed distinct functions. Compared with EC2 which highly expressed surface adhesion proteins such as ITGA1/B1, EC1 secreted a variety of chemokines, leading to the infiltration of macrophages. The presence of Myo-FBs incapacitated the function of Nor-FBs, as Myo-FBs secreted less variety of collagen. Myo-FBs also lost the function of vegetating CMs for downregulating PTN-PTPRS pair. Although we can’t clearly identify the function of each ligand-receptor pairs this new microenvironment must be critical to myocardial fibrosis and weakened contractility of the heart (Figure 7C) which was the main reason for HF.

## 4. Discussion

In our present study, we observed a holistic transformation of cardiac cells (from normal, cHF, and dHF human samples). Previously unrecognized phenotypes in HF samples were identified in our study, which may contribute to reduced myocardial contractility. CMs, which act as the core cells of myocardial contraction, play an important role in HF progression through energy metabolism reduction and abnormal expression of myosin subunits. In addition, our study uncovered a host of previously unrecognized functions in ECs and FBs, for example, Nor-FBs can transform into Myo-FBs, accompanied by decreased collagen secretion and increased expression of muscle proteins. These results are consistent with previous findings that functional transformation of multiple cells can aggravate HF [23,24]. In summary, it is found that: (i) three kinds of cardiac cells underwent phenotypic transformation, including CMs, ECs, and FBs; (ii) the expression of myosin subunits in cCM is abnormal, including the increased expression of MYH7, and decreased expression of *MYH6*, *MYL4*, *MYL7*; (iii) ECs have two specific disease-related phenotypes in HF, named inflammatory phenotype and muscularized phenotype; And (iv) Myo-FBs are increased in HF and are highly associated with cardiac fibrosis. Although we can’t exactly control the construction of samples since the data comes from the GEO dataset, the single-cell sequence profile of human failure heart can accurately demonstrate the heterogeneity of cardiac cells in HF, which contributes to understanding the mechanism of development of HF. Our primary findings firstly highlighted the important involvement of phenotypic transformation of cardiac cells in HF induced by coronary heart disease and dilated cardiomyopathy.

Reduced contractility of CMs and myocardial fibrosis are considered to be the main causes of HF, mainly due to a decrease in the number of normal CMs and abnormal extracellular matrix construction [25]. It was found that the phenotypic transformation of CMs appeared in HF, accompanied by functional and transcriptional shift, leading to reduced contractility. Pseudotime trajectory analysis has been widely applied in many diseases to predict cell differentiation trajectory, so as to simulate the process of cell dynamic changes [26]. Given that, our data suggested that CMs differentiated into specific phenotypes, which represented a major factor of decreased cardiac function. Using scRNA-seq, it was found that decreased energy metabolism both existed in cCM and dCM, indicating the mitochondrial dysfunction in CMs. Moreover, dCM had lower energy metabolism than cCM. Mitochondrial dysfunction has been widely known in ischemic myocardium, which can cause cardiac insufficiency. Ikeda.et al.[27] put forward that autologous mitochondrion and their related energy source transferred by extracellular vesicle could enhance cardiac function of ischemic myocardium, including HF, through restoration of myocardial bioenergetics. In addition, we noticed that dCM presented much more severe mitochondrial dysfunction, accompanied by T cell infiltration. Chronic myocarditis and immune infiltration are important reasons for dilated cardiomyopathy [28]. Studies have suggested that T helper cell 17 (Th17), which constituted the main T cell population, can aggravate inflammation [29]. Our results also demonstrated that dCM was highly reactive to T cells, compared to cCM. Taken together, we assumed that T cell infiltration may damage mitochondrial function, leading to a severe abnormal energy metabolism, which was common in some immunotherapy of tumor.

DEGs between NCM and cCM suggested that myosin subunits were dramatically changed in cHF, including MLC and MHC. In MHC, the ratio of *MYH6* to *MYH7 i*n cCM was inversed, which had been verified to cause reduced myocardial contractility, because *MYH6* had higher actin-binding ability and actin-activation ATPase activity than *MYH7* [30,31,32]. In MLC, we first paid attention to the decreased expression of *MYL7*, which was the RLC of myosin. As is well known, the function of RLC to hydrolyze ATP for releasing energy is important to myocardial contractility. Given that, the downregulation of *MYL7* indicated impaired ability of CMs to release energy by hydrolyzing ATP [11]. Targeted therapies have been proposed by increasing RLC activation. Omecamtiv mecarbil (OM), a novel drug with selective activation of myocardial myosin has been applied into clinical practice [33]. OM can reduce the mortality in HF patients with reduced ejection fraction by increasing myosin ATPase ratio [34,35]. Furthermore, OM has no adverse effect on blood pressure, ventricular arrhythmia, potassium concentration or kidney function compared to other commonly used HF drugs. This evidence suggests that myosin-targeted therapy is feasible. Taken together, we considered that therapy which targets increased expression of RLC combined with restoration of mitochondria would be a promising treatment for cHF. In addition, although the functions of MYL4 in cHF were poorly understood currently, variants of MYL4 have been shown as a causative factor in certain cardiomyopathy, especially in hypertrophic cardiomyopathy [36,37]. The characteristic of dCM as well as the mechanism of dilated cardiomyopathy were clearly clarified; however, the mutation of myosin subunits, which has been considered as the important reason of dilated myocardiopathy, was hardly observed due to the limitations of scRNA-seq technology. Therefore, we didn’t exclude the possibility that the structural changes of myosin existed in dCM.

After demonstrating that ECs derived from the ventricle and atrium had similar properties, we proposed that ECs presented three distinct phenotypes in HF, and defined them as inflammatory ECs, muscularized ECs, and normal ECs. These three phenotypes showed different gene expression patterns and pathway enrichments. There is evidence that inflammatory ECs are involved in many cardiovascular diseases, such as atherosclerosis and rheumatic endocarditis [38]. Besides, a previous study has confirmed that immunity infiltration can lead to cardiac remodeling [39]. Angiotensin-converting-enzyme inhibitors (ACEIs) is a classical drug therapy after myocardial infarction (MI) with the role of inhibiting cardiac remodeling and improving prognosis of HF. Long-term employment of ACEIs can limit vascular inflammation, particularly the infiltration of monocytic series, and reduce peroxidation damage, resulting in improved endothelial function in mice with ischemic HF [40]. Collectively, our study also demonstrated that the inflammatory ECs can recruit and interact with immunity cells. Muscularized ECs is a specific phenotype that we firstly identified in HF. To exclude the possibility of the cytomixis of CMs and ECs in data, immunofluorescence was further applied, and the results showed that vascular endothelial cells in HF rats actually highly expressed *MYH6* and *MYL7*, confirming the appearance of muscularized ECs. Muscularized ECs have been proposed previously in other diseases, for example, muscularization of intrapulmonary arterioles due to hypoxia can lead to pulmonary arterial hypertension (PAH), while inflammation and dysfunction of endothelial are considered two primary instigators [41]. PAH shows reduced blood flow to the lungs, leading to the exacerbation of hypoxia, which is similar to the pathological process in cHF. It was suspected that the presence of muscularized ECs can cause arterioles muscularization in HF, which may affect the blood supply and aggravate myocardial systolic disorders. However, this special phenotype provided an opportunity to develop novel therapies for cell transplantation. The injection of ECs and targeted conversion into muscularized endothelial cells may be an effective and promising approach for the treatment of cardiac reconstruction.

FBs, which can synthesize and secrete various kinds of collagen to form the extracellular matrix, exert enormous functions on supporting and maintaining the structural integrity of the heart after myocardial infarction. Myo-FBs is a special type of FBs that contains actin, myosin, and other muscle proteins. Its continuous activation can lead to myocardial fibrosis, resulting in poor myocardial remodeling [32,42]. In our study, Myo-FBs were significantly increased and the function of secreting collagen to form extracellular matrix was reduced in HF. The transformation from Nor-FBs to Myo-FBs in our study demonstrated that Myo-FBs played a certain part in myocardial fibrosis. In addition, we suspected that inhibiting the continuous activation of Myo-FBs may have the potential to prevent the deterioration of HF.

Functional experiments were applied in our study to confirm the sequencing results, but there are still some inadequacies. Therefore, the gene expression pattern, pathway enrichment, and regulation network were used to demonstrate the functional transformation of cardiac cells in the process of HF as a replacement. Further research will be applied to support our conclusion through more functional experiments.

Collectively, our study accurately defined the phenotypic transformation of three main cardiac cells and highlighted the functional shift of cardiac cells in HF progression. These results provided a framework to demonstrate changes in gene expression with the pathophysiological state. It was suspected that these specific pathophysiological stage phenotypes were the core mechanisms of HF. Furthermore, an integrated map about the communication among different phenotypes in cHF was exhibited. Our work can serve as a reference for studies about the mechanisms underlying the HF progression as well as providing a powerful tool to discover novel drug targets for intervention.

## Data Availability

ScRNA sequence profile was download from the GEO database (GSE121893 and GSE109816). Array of expression profile was download from the GEO database (GSE42955).

## Supplementary Materials

The following supporting information can be downloaded at: https://www.mdpi.com/article/10.3390/biomedicines10020402/s1, Figure S1: Five cell types were identified from single-cell transcriptome of heart disease; Figure S2: Cardiomyocytes (CMs) cluster into 4 distinct phenotypes; Figure S3: Endothelial cells in HF have two special disease-related phenotypes; Figure S4: Fibroblasts transformed into myofibroblasts.

## Abbreviations

HF	Heart Failure
scRNA-seq	Single-cell RNA sequencing
UMAP	Unified Manifold Approximation and Projection
CMs	Cardiomyocytes
ECs	Endothelial Cells
FBs	Fibroblasts
MPs	Macrophages
SMCs	Smooth Muscle Cells
Nor-FBs	Normal fibroblasts
Myo-FBs	Myofibroblasts
cHF	HF caused by coronary heart disease
dHF	HF caused by dilated cardiomyopathy
nHF	Normal heart without HF
cCM	Cardiomyocyte derived from cHF
dCM	Cardiomyocyte derived from dHF
NCM	Normal cardiomyocyte
TCM	Transient cardiomyocyte
MLC	Myosin light chain
MHC	Myosin heavy chain
RLC	Regulatory Light Chain
ELC	Essential Light Chain
GEO	Gene Expression Omnibus
DEGs	Differentially Expressed Genes
Padj	Adjusted P value
TFs	Transcription Factors
GSVA	Gene Set Variable Analysis
GSEA	Gene Set Enrichment Analysis
SCENIC	Single-Cell Regulatory Network Inference and Clustering
LVPWs	Left Ventricular Systolic Posterior Wall Thickness
LVIDs	Left Ventricular Systolic Inner Diameter
EDV	End Diastolic Volume
LVPWd	Left Ventricular Diastolic Posterior Wall Thickness
LVIDd	Left Ventricular Diastolic Inner Diameter
ESV	End-Systolic Volume
FS	Fractional Shortening
EF	Ejection Fraction
