# Supplementary material for: Cellular Phenotypic Transformation in Heart Failure Caused by Coronary Heart Disease and Dilated Cardiomyopathy: Delineating at Single-Cell Level
Source: Biomedicines. 2022 Feb 8;10(2):402. doi: 10.3390/biomedicines10020402 (PMC8962334; doi:10.3390/biomedicines10020402)
Supplement: Supplementary file 1 [file biomedicines-10-00402-s001.zip › biomedicines-1559796-supplementary.pdf]

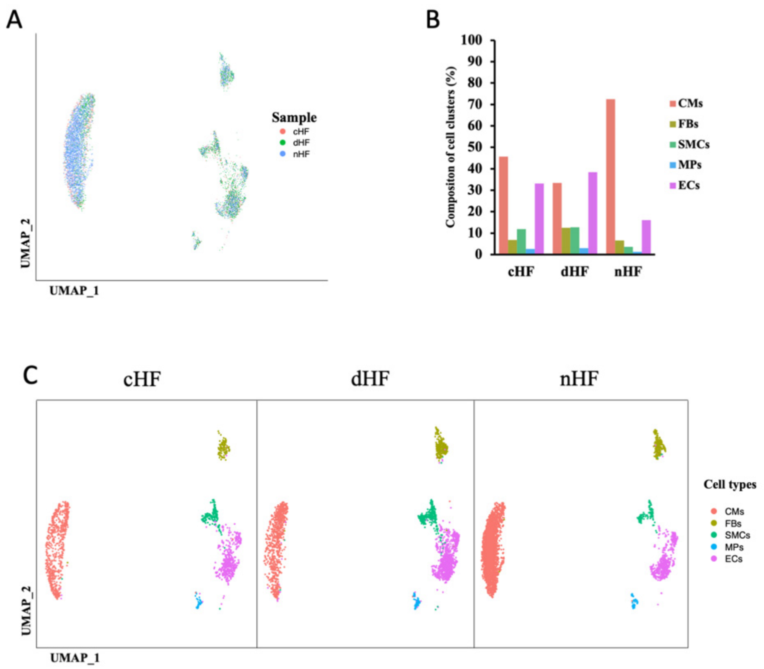

Supplementary Figure S1. Five cell types were identified from Single-cell transcriptome of heart disease. (A) UMAP plots of five cell types, clusters are color-coded by cell sample. **(B)** proportion of five cell types in each sample. **(C)** UMAP plot of each sample (cHF, dHF and nHF), colored by cell types.

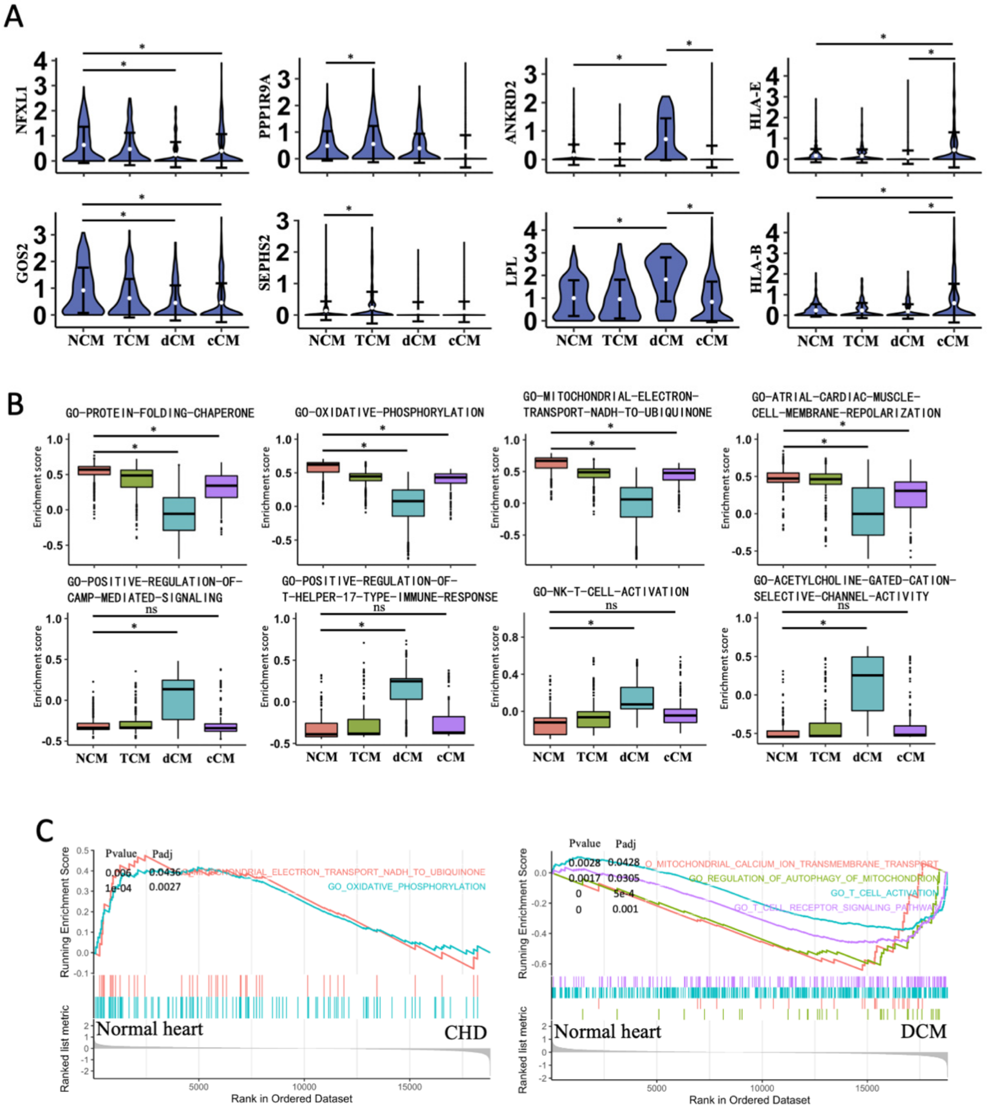

Supplementary Figure S2. Cardiomyocytes (CMs) cluster into 4 distinct phenotypes. (A) violin plots indicate differentially expressed gene in phenotypes of CMs. *Padj < 0.05 Wilcox test. **(B)** Multiple pathway enrichments show the characteristics of the cCM and dCM, as well as the difference between cCM and dCM. Boxplot y axis demonstrates enrichment score of pathways. *padj < 0.05 Wilcox test. **(C)** GSEA results showed the pathway difference between normal heart and ischemic or diluted cardiomyopathy.

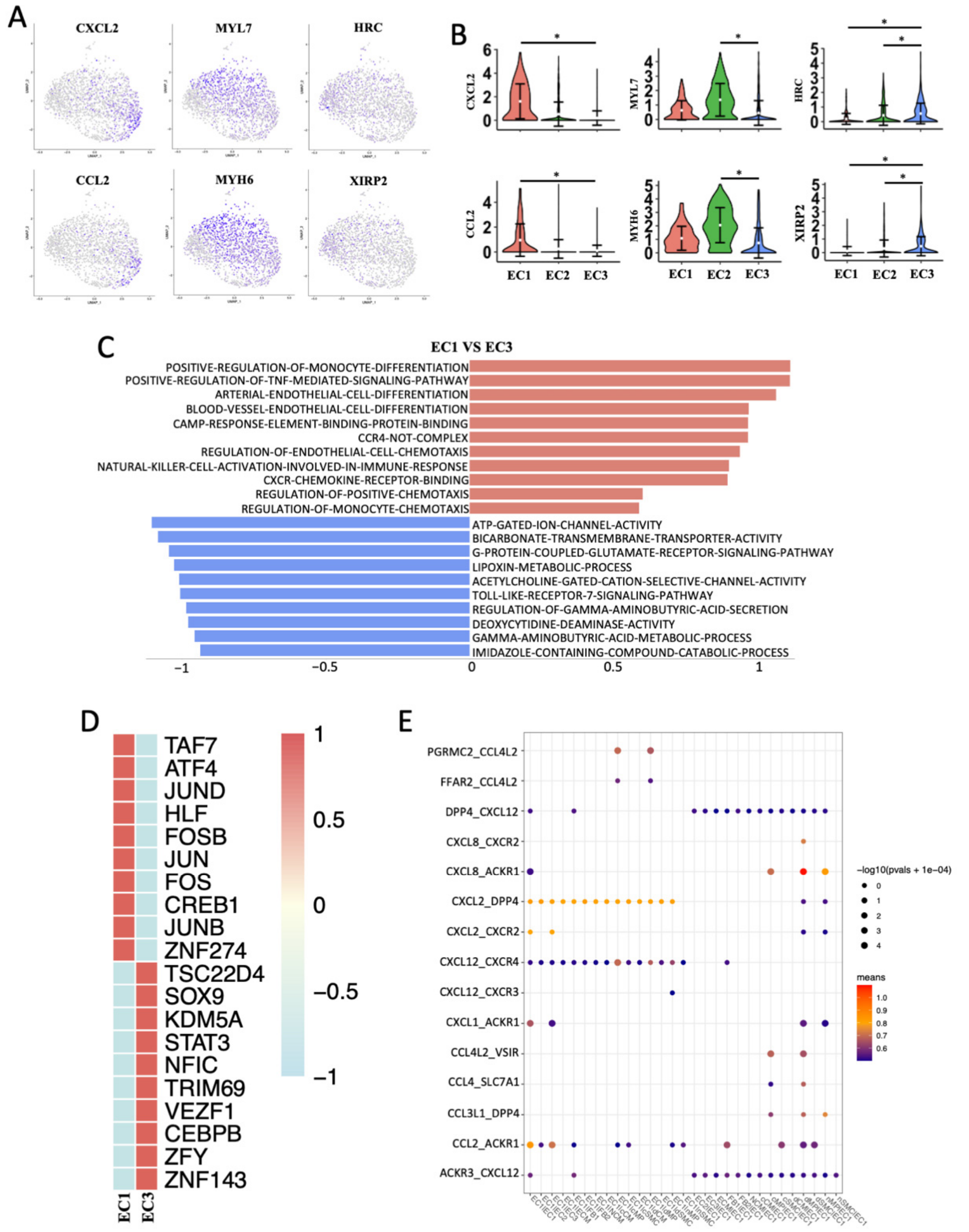

Supplementary Figure S3. Endothelial cells in HF have two special disease-related phenotypes. (A) Expression of marker genes for the phenotypes of ECs defined above each panel. **(B)** The expression of known cell type discriminating genes in EC1, EC2 and EC3 from 3 samples. *Padj < 0.05 Wilcox test. **(C)** Differences in pathway

enrichment scored by GSVA between EC1 and EC3. **(D)** Differences in TFs active scored by SCENIC between EC1 and EC3. **(E)** Immunity-related interacting pair between EC1 and other cell clusters.

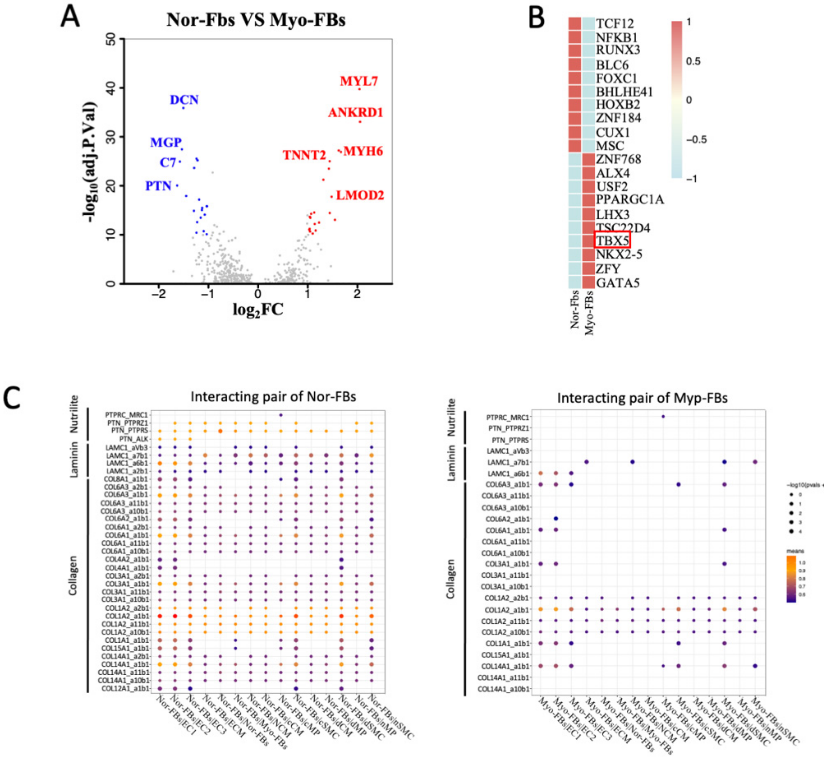

Supplementary Figure S4. Fibroblasts transformed into Myofibroblasts. (A) Volcano show the DEGs ($log_2FC>1$) between Nor-FBs and Myo-FBs. **(B)** Differences in TFs active scored by SCENIC between Nor-FBs and Myo-FBs. **(C)** Crosstalk of Nor-FBs or Myo-FBs showed the functional change during the phenotypic transformation.
